# Crosslinked PEG and PEBAX Membranes for Concurrent Permeation of Water and Carbon Dioxide

**DOI:** 10.3390/membranes6010001

**Published:** 2015-12-23

**Authors:** Colin A. Scholes, George Q. Chen, Hiep T. Lu, Sandra E. Kentish

**Affiliations:** Peter Cook Centre for Carbon Capture and Storage Research, Department of Chemical & Biomolecular Engineering, The University of Melbourne, Melbourne VIC 3010, Australia; cascho@unimelb.edu.au (C.A.S.); gechen@unimelb.edu.au (G.Q.C.); h.lu6@student.unimelb.edu.au (H.T.L.)

**Keywords:** carbon dioxide capture, poly ethylene glycol, PEBAX, carbon dioxide, water, Lennard Jones, solubility

## Abstract

Membrane technology can be used for both post combustion carbon dioxide capture and acidic gas sweetening and dehydration of natural gas. These processes are especially suited for polymeric membranes with polyether functionality, because of the high affinity of this species for both H_2_O and CO_2_. Here, both crosslinked polyethylene glycol diacrylate and a polyether-polyamide block copolymer (PEBAX 2533^©^) are studied for their ability to separate CO_2_ from CH_4_ and N_2_ under single and mixed gas conditions, for both dry and wet feeds, as well as when 500 ppm H_2_S is present. The solubility of gases within these polymers is shown to be better correlated with the Lennard Jones well depth than with critical temperature. Under dry mixed gas conditions, CO_2_ permeability is reduced compared to the single gas measurement because of competitive sorption from CH_4_ or N_2_. However, selectivity for CO_2_ is retained in both polymers. The presence of water in the feed is observed to swell the PEG membrane resulting in a significant increase in CO_2_ permeability relative to the dry gas scenario. Importantly, the selectivity is again retained under wet feed gas conditions. The presence of H_2_S is observed to only slightly reduce CO_2_ permeability through both membranes.

## 1. Introduction

Post combustion carbon dioxide capture requires the removal of carbon dioxide from flue gas streams saturated with water. Membrane technology is considered of potential for this application because of its smaller lighter footprint, relative to solvent absorption, but comparable costs [1,2,3]. In this application, co-permeation of water vapour with the CO_2_ can be advantageous, as it dilutes the permeate stream, reducing the CO_2_ partial pressure and thus increasing the driving force for CO_2_ permeation [2].

Similarly, two major unit operations in natural gas processing are gas sweetening, which removes carbon dioxide and hydrogen sulphide; and gas dehydration which removes water [4]. This ensures the natural gas composition is standardised for transport and limits the risk of water hydrates and pipeline corrosion. Membrane gas separation is increasingly being used for natural gas sweetening [5,6,7,8,9,10]. Dehydration of natural gas is traditionally undertaken through an ethylene glycol process [11]; however polysulfone membranes have been used for this application [12]. There is the potential to combine both natural gas sweetening and dehydration in a single membrane process, although care would be required to ensure pressure conditions are suitable to avoid both methane and carbon dioxide hydrates; and to avoid corrosion in the permeate stream piping.

To undertake either of these operations, the membrane needs to have high permeability for water, and CO_2_, while also having good selectivity against other gases. Water permeability through any polymeric membrane is usually orders of magnitude greater than other gases and vapors, due to the small size and high critical temperature of this molecule. Membranes containing polyether groups are of particular interest for CO_2_ removal, due to the strong interaction between the quadripolar CO_2_ and the polar ether bonds [13,14,15]. However, crystalline regions within the membrane can reduce the permeability of such gas species, due to the reduction in fractional free volume within these regions. Pure polyethylene glycol (PEG) membranes suffer significantly from such crystallinity, with values reported of up to 71 vol% [16]. The use of cross linking, or the incorporation of polyamide blocks within the structure, as commercialised through the PEBAX^©^ series of block copolymers, reduces the overall crystallinity, with values of 14% to 51% reported for PEBAX systems [17].

In this investigation, the performance of cross-linked PEG and PEBAX 2533 in single and mixed gas feeds of CO_2_ and water are reported. In particular, the effect of competitive sorption on the permeability of CO_2_, water, N_2_, CH_4_ and H_2_S is studied, to identify the potential of these two polymeric membranes for post combustion carbon capture and for simultaneous removal of both acid gases and water from natural gas.

## 2. Experimental

PEBAX 2533 is a block copolymer of 80 wt% poly(tetramethylene oxide) and 20 wt% polyamide (Nylon 12) and 14% crystallinity in the polyamide block [17]. The polymer was supplied by Arkema (Melbourne, Australia), and cast as flat sheet membranes from 1-butanol (3 wt%). Crosslinked PEG was synthesized from poly (ethylene glycol) dimethyl ether acrylate, M_n_ 454 g/mol (Aldrich, Sydney, Australia) and 1-hydroxyl-cyclohexyl phenyl ketone (Irgacure 184) with 50% water, and cast as flat sheet membranes. Cross-linking was promoted through UV light, following the procedure established by Lin and Freeman [18]. Both polymeric membranes where dried in a vacuum oven at 80 °C for 12 h, producing membranes of average thickness ~70 μm. Pure CO_2_ (industrial grade), CH_4_ (high purity), N_2_ (high purity), Ar (ultrahigh purity), He (ultrahigh purity), a 90% CH_4_—10% CO_2_ mixture, 90% N_2_—10% CO_2_ mixture as well as a 90% N_2_—10% CO_2_ mixture with 500 ppm H_2_S were supplied by BOC Gas Ltd (Melbourne, Australia).

Sorption measurements were conducted on a Gravimetric Sorption Analyzer (GHP-FS, VTI Scientific Instruments, Irvine, CA, USA), with a Cahn D-200 microbalance, described elsewhere [19]. For CO_2_, CH_4_ and N_2_ isotherms the analyzer was operated in static mode. The membranes were initially exposed to vacuum overnight and heated to 80 °C to remove air and water vapour. The penetrant gas was introduced into the chamber in pressure steps at 35 °C and the equilibrium mass of membrane plus sorbed gas was measured. In an incremental manner, penetrant isotherms as a function of penetrant pressure were determined. Sorption mass equilibrium for all gases was reached within a maximum of 6 h at each pressure. A comparable experiment using helium was completed to determine the buoyancy correction. For water sorption, the Gravimetric Sorption Analyzer was operated in a flow mode. The standard degassing procedure as above was undertaken. Helium, the carrier gas, was then introduced into the sample chamber at a set pressure, ~1 atm, in a flow through arrangement. The helium source was divided into two streams, one dry, the other passed through a water entrainer at a set temperature. Relative humidity within the sample chamber was achieved by varying the flowrate ratio of these two streams. Sorption mass equilibrium was achieved within 3 h at each relative humidity stage.

Single gas permeabilities were measured on a constant volume, variable pressure gas permeation apparatus described elsewhere [20]. Mixed gas permeabilities were tested on a mixed gas instrument, also reported elsewhere [21]. The effect of humidity was measured on a modified mixed gas permeability instrument reported elsewhere [13]. Pure gas or a gas mixture (10% CO_2_ in CH_4_) was fed into a saturator vessel filled with water, then a demister vessel to generate the humid gas stream. Both the saturator and demister were partially filled with steel wool and immersed in a temperature controlled bath. The wet gas stream was then passed through a humidity and temperature transmitter (HMT, Probe type 334 Vaisala Oyj, Vantaa, Finland), which was fitted within a fan forced oven. The oven also contained the permeation cell, another humidity sensor (HMT, Vantaa, Finland) on the permeate side and associated tubing. Stainless steel wool packing was present in both sides of the membrane within the permeation cell, to enhance mixing and minimize concentration polarization. Before each series of experiments, the permeation cell was pre-heated to the operating temperature for at least an hour, with nitrogen and argon flowing through both sides of the membrane, to avoid vapor condensation during the experiment. The humidity of the feed stream was controlled by the temperature of the saturator and the demister. The permeate side of the membrane used argon as the sweep gas and after the humidity sensor passed through an iced cold trap to remove water. All permeabilities were measured at 35 °C for a total upstream pressure of 600 kPa gauge. For mixed gas measures, the concentrations of gases in the permeate stream were determined by gas chromatography (Varian CP-3800, column PORAPAK Q in a bypass series with a Molecular Sieve 5A) (Varian, Melbourne, Australia).

## 3. Results and Discussion

**Figure 1:**
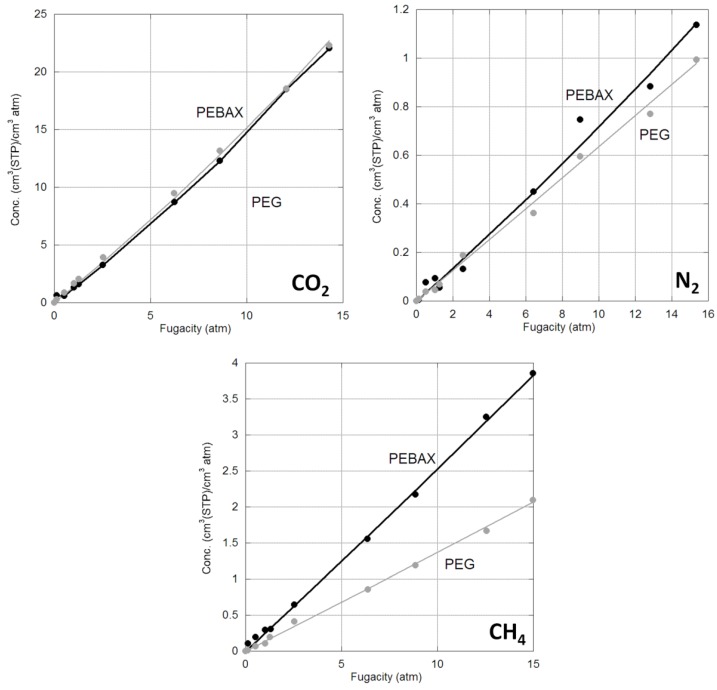
CO_2_, N_2_ and CH_4_ sorption isotherms in PEBAX (black) and PEG (grey) at 35 °C.

**Figure 2:**
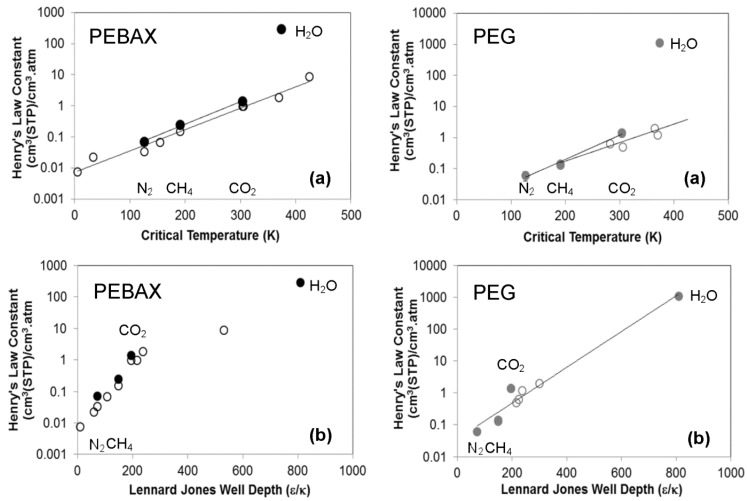
Correlation between the infinite dilution solubility or Henry’s Law constant at 35 °C and the (**a**) Critical Temperature and (**b**) Lennard Jones Well Depth (ε/κ) of a range of penetrants for PEBAX(black) and PEG (grey). Filled symbols are the data from this work, while the open symbols are data from Bondar *et al.* [17] (PEBAX) and Lin and Freeman [18] (PEG).

The sorption isotherms for CO_2_, N_2_, CH_4_ in both crosslinked PEG and PEBAX membranes are provided in Figure 1. The isotherms are linear and can be described by Henry’s Law, consistent with the literature for small gases in rubbery polymers at low pressure [14]. The Henry’s law constant for CO_2_, N_2_ and CH_4_ can be determined from the concentration isotherms based on fugacity and are compared to literature values in Table 1 and Figure 2. The data for crosslinked PEG is consistent with the literature, while that for PEBAX 2533 is slightly higher.

**Table 1:** Henry’s law constants for pure gases in crosslinked PEG and PEBAX 2533 at 35 °C.

Gas or Vapor	Fundamental Properties		Henry’s Law Constant (cm^3^/cm^3^·atm)			
	Critical Temperature T_c_ (K)	Lennard Jones Well Depth (ε/κ) [22]	Crosslinked PEG This Work	Crosslinked PEG [23]	PEBAX 2533 This Work	PEBAX 2533 [17]
CO_2_	304.21	213.4	1.39 ± 0.20	1.5 ± 0.1	1.39 ± 0.20	0.963
N_2_	126.2	83	0.06 ± 0.02	–	0.07 ± 0.02	0.0334
CH_4_	191.05	154.7	0.14 ± 0.03	0.14 ±0.02	0.25 ± 0.05	0.152
H_2_O	373.95	809.1	1100 ± 200	–	290 ± 50	–

**Figure 3:**
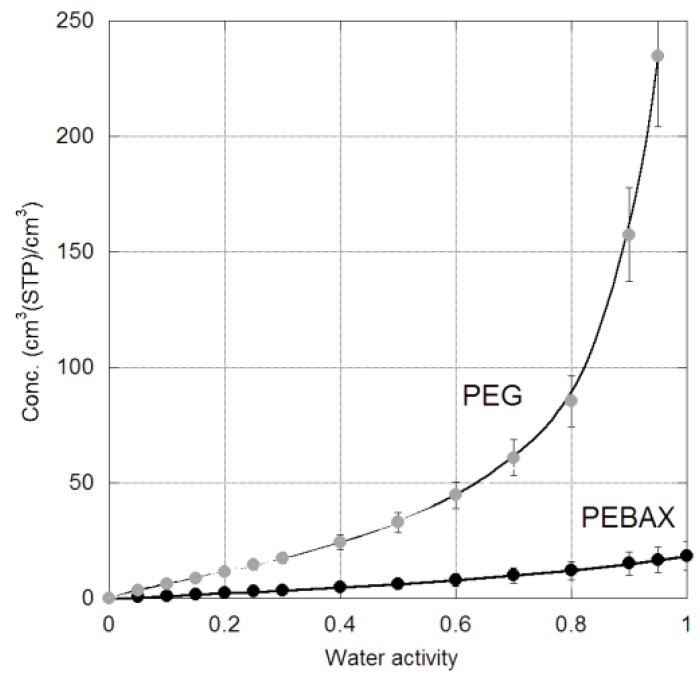
H_2_O sorption isotherm in PEBAX (black) and PEG (grey) at 35 °C.

The H_2_O isotherm for both membranes (Figure 3) is different from the other gases in that it is strongly concave at high H_2_O activity, especially for the crosslinked PEG system. This behaviour is typical of highly condensable vapors [24] and the PEG result at high activity suggests extensive swelling of the polymer and the formation of a hydrogel, as commonly observed in the literature [25]. This represents a significant morphology change from the dry membrane, as the hydrogel membrane swells and elasticity increases dramatically. The water solubility in PEBAX is lower due to the presence of the “hard” polyamide domains, which do not swell as readily in water [26]. Sophisticated models such as PC-SAFT [27,28] or the ENSIC model [29] are required to describe the swelling effects that are evident at higher water activity, but this is outside the scope of this paper. However, the Henry’s Law constant can be estimated from the data at low water activity (the infinite dilution solubility) and these values are also provided in Table 1 and Figure 2.

Generally, it is found that such Henry’s Law coefficients can be correlated to either the Lennard-Jones well depth [17] of the gas or to its critical temperature [18]. Either approach is able to fit the data here for CO_2_, N_2_ and CH_4_, but the Lennard Jones approach is significantly more consistent with the results for water. The use of the critical temperature as the correlating parameter leads to a substantial under-estimation of this parameter (Figure 2).

The pure gas permeability in crosslinked PEG and PEBAX 2533 for CO_2_, N_2_ and CH_4_ at 35 °C are provided in Table 2. Both PEG and PEBAX clearly favour CO_2_ over N_2_ and CH_4_. For PEG, the N_2_ and CH_4_ permeability is reduced compared to literature, while the CO_2_ permeability is half that reported in the literature (130 Barrer) [23,30]. This suggests that the level of crystallinity has not been reduced to the same extent as that observed by Lin *et al.* [23]. This is associated with the level of cross-linking and the difference in the acrylic length of the original polymer influencing membrane density and fractional free volume. However, the CO_2_ permeability is around five times greater than that of semicrystalline (uncrosslinked) PEG of 12 Barrer [16].

**Table 2:** Single gas permeability (Barrer) and ideal selectivity through PEG and PEBAX membranes at 35 °C and 600 kPa.

Gas	PEG	PEBAX
CO_2_	66 ± 2	212 ± 5
N_2_	1.6 ± 0.1	6.4 ± 0.2
CH_4_	4.2 ± 0.1	29.5 ± 0.4
CO_2_/N_2_ Selectivity	41	33
CO_2_/CH_4_ Selectivity	16	7.2

For PEBAX 2533, the CO_2_ permeability is comparable to Bondar *et al.* [17] who obtained a value of 221 Barrer for membranes prepared by melt extrusion. Tocci *et al.* [31] report a slightly lower CO_2_ permeability of 200 Barrer and a slightly higher CH_4_ value of 40 Barrer for a membrane formed from a n-butanol/2-propanol solution and annealed at 70 °C for 24 h. Barbi *et al*. [26] show that the permeability value is indeed strongly dependent upon the casting solvent and annealing conditions. They record values of 276 Barrer (CO_2_) and 40 Barrer (CH_4_) when the membrane is cast in 1-butanol and 241 Barrer (CO_2_) and 35 Barrer (CH_4_) when it is cast in cyclohexanol; with annealing in both cases at 70 °C for 12 h. They argue that membranes cast from cyclohexanol exhibit a decreased “hard” (polyamide) domain fraction due to an imperfect phase separation.

In terms of selectivity, PEBAX has lower CO_2_/N_2_ and CO_2_/CH_4_ selectivity compared to PEG. These differences are believed to be due to the underlying morphology of the polymers. PEBAX 2533 has a lower density (1.00 g/cm^3^) than the cross-linked PEG (1.31 g/cm^3^), and as such the more open morphology of PEBAX enabled higher gas permeabilities. However, the more open morphology is at the expense of selectivity, which is a common trade-off for polymeric membranes [32].

**Figure 4:**
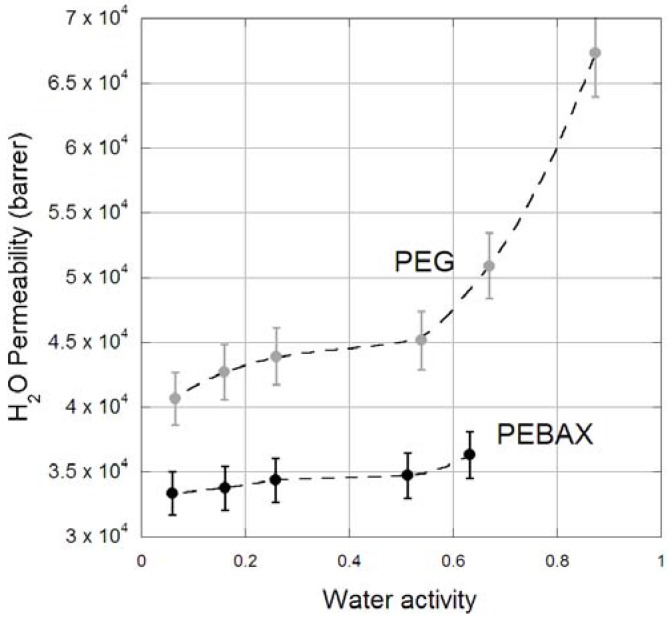
Water Permeability (Barrer) in PEBAX (black) and PEG (grey) membranes within a humidified N_2_ feed gas stream.

The H_2_O permeability through PEG and PEBAX membranes as a function of feed humidity is provided in Figure 4, with N_2_ carrier gas. The PEBAX H_2_O permeabilities are of the same order of magnitude as reported in the literature; Sijbesma *et al.* [33] reported a H_2_O permeability of ~25,000 Barrer, Gugliuzza and Drioli [34] reported 25,030 Barrer, while Rezac *et al.* [35] reported 25,600 for a water activity of 0.53. Our own values are slightly higher, possibly as a result of the care we have taken to eliminate concentration polarization. Conversely, the H_2_O permeabilities for PEG reported here are lower than those in the literature for copolymer series based on PEG [36,37]. This difference is attributed to the cross-linking in this PEG membrane, decreasing water diffusion within the resulting membrane and hence reducing water permeability. For both membranes the H_2_O permeability increases as a function of relative humidity, which is observed for other polymeric membranes and is indicative of water swelling. For PEG, a significant increase in permeability occurs above an activity of 0.6, suggestive of a transition in the membrane morphology to a hydrogel. This observation has also been observed for other PEG based membranes [36,37]. The average H_2_O/N_2_ ideal selectivity of PEG is 28,000 and for PEBAX is 5400, which is comparable to other rubbery polymeric membranes [33].

The permeability of CO_2_ for PEG and PEBAX in dry N_2_-CO_2_ and CH_4_-CO_2_ mixed gas conditions are shown in Table 3. In both membranes and both mixed gas systems the observed permeability of CO_2_ is reduced compared to the pure gas permeability, because of competition from the other gas. For both N_2_-CO_2_ and CH_4_-CO_2_ feeds, the CO_2_ permeability in PEG is reduced by 11% and for PEBAX by 10%, compared to the single gas case. For N_2_ and CH_4_, the average permeability through both membranes is also reduced, due to competitive sorption from CO_2_. As such, the CO_2_/N_2_ and CO_2_/CH_4_ selectivities of both membranes are slightly lower compared to the single gas measurements, but both still retain significant selectivity for CO_2_.

**Table 3:** Gas permeability (Barrer) through PEG and PEBAX membranes under dry mixed gas conditions at 35 °C and 600 kPa.

Gas Mixture	Gas	PEG	PEBAX
90% N_2_—10% CO_2_	CO_2_	59 ± 0.4	191 ± 0.8
	N_2_	1.5 ± 0.1	6.2 ± 0.2
	CO_2_/N_2_	39	31
90% CH_4_—10% CO_2_	CO_2_	59 ± 0.4	191 ± 0.9
	CH_4_	4.1 ± 0.2	28 ± 0.3
	CO_2_/CH_4_	14	7

A water activity of 0.2 is then applied to the CH_4_-CO_2_ feed gas to simulate wet conditions in a standard natural gas process [6]. For both PEG and PEBAX the CO_2_ permeability changed little compared to the dry mixed gas (Table 4). Similarly, the CH_4_ permeability increased only slightly for both PEG and PEBAX compared to the dry mixed gas feed. As a result, the ideal CO_2_/CH_4_ selectivity of both membranes decreased only slightly. Hence, CO_2_ separation performance is retained under wet feed conditions, indicating both membranes could process a low RH feed gas without a loss in performance.

**Table 4:** Gas permeability (Barrer) through PEG and PEBAX membranes under different mixed gas conditions at 35 °C.

Gas Mixture	Gas	PEG	PEBAX
90% CH_4_—10% CO_2_ with 20% RH	CO_2_	60 ± 0.5	194 ± 1.0
	CH_4_	4.2 ± 0.4	29.1 ± 0.5
	H_2_O	42,400 ± 2500	36,000 ± 2100
	CO_2_/CH_4_	14	7
90% N_2_—10% CO_2_ with 500 ppm H_2_S	CO_2_	58 ± 0.4	189 ± 0.8
	N_2_	1.3 ± 0.2	6.0 ± 0.4
	CO_2_/N_2_	45	32

The water permeability through PEBAX also appears to increase slightly under the mixed gas conditions; compared to the pure N_2_ feed gas measurement (Figure 3) at the same activity, although the increase is possibly within experimental error. This increase is attributed to CO_2_ inducing plasticization in the PEBAX structure and thus increasing the water diffusivity through the membranes. The water permeability through PEG is within error of the N_2_ feed gas result at the same activity (Figure 3).

Under dry feed gas conditions adding 500 ppm H_2_S to the N_2_-CO_2_ mixed gas system enables the impact of H_2_S on separation performance to be measured, with the change in CO_2_ and N_2_ permeability reported in Table 4. For PEG, the presence of H_2_S reduces CO_2_ permeability by 1 Barrer for PEG and 2 Barrer for PEBAX. These are only minor decreases in performance and signify that H_2_S competition for sorption in both of these membranes is small. When compared to that previously reported for PDMS exposed to H_2_S (~8% reduction observed [38]), it highlights the different chemical interactions between the gases and moieties in the two polymers studied here. Similarly, only a small change in N_2_ permeability upon exposure to H_2_S for both PEG and PEBAX was observed. Hence, the CO_2_/N_2_ selectivity of both membranes in the presence of 500 ppm H_2_S is retained.

## 4. Conclusions

Membranes consisting of cross-linked PEG and PEBAX 2533 where studied for CO_2_ and H_2_O separation from CH_4_ and N_2_, to simulate applications including post-combustion carbon capture and natural gas sweetening and dehydration. Sorption measurements indicated that the simple gases followed Henry’s law within both membranes while water sorption increased substantially at higher water activities, particularly for the PEG membrane. The Henry’s Law coefficients were more readily fitted to a correlation with the Lennard Jones well depth than to the critical temperature. Single gas measurements indicated that both membranes were selective for CO_2_ against CH_4_ and N_2_, while water permeability increased strongly associated with water activity in the feed gas. CO_2_ permeability fell in mixtures with CH_4_ and N_2_, compared to the single gas measurement, because of competitive sorption. Under wet feed gas conditions, the presence of water swelled both membranes and the gas permeability increased, with only a small decrease in selectivity. It was also observed that H_2_S had very little impact on both PEG and PEBAX membranes separation of CO_2_ from N_2_.
